# A Review on Electrospun Nanofiber Composites for an Efficient Electrochemical Sensor Applications

**DOI:** 10.3390/s23156705

**Published:** 2023-07-26

**Authors:** Ramkumar Vanaraj, Bharathi Arumugam, Gopiraman Mayakrishnan, Ick Soo Kim, Seong Cheol Kim

**Affiliations:** 1 School of Chemical Engineering, Yeungnam University, Gyeonsan 38541, Republic of Korea; ramkumar@yu.ac.kr (R.V.); arvbk_11@yu.ac.kr (B.A.); 2 Nano Fusion Technology Research Group, Division of Molecules and Polymers, Institute for Fiber Engineering (IFES), Interdisciplinary Cluster for Cutting Edge Research (ICCER), Shinshu University, Tokida 3-15-1, Ueda 386-8567, Nagano, Japan; gopiraman@shinshu-u.ac.jp

**Keywords:** Electrospun, metal oxide, carbon fiber, surface modification, selectivity, sensors

## Abstract

The present review article discusses the elementary concepts of the sensor mechanism and various types of materials used for sensor applications. The electrospinning method is the most comfortable method to prepare the device-like structure by means of forming from the fiber structure. Though there are various materials available for sensors, the important factor is to incorporate the functional group on the surface of the materials. The post-modification sanction enhances the efficiency of the sensor materials. This article also describes the various types of materials applied to chemical and biosensor applications. The chemical sensor parts include acetone, ethanol, ammonia, and CO_2_, H_2_O_2_, and NO_2_ molecules; meanwhile, the biosensor takes on glucose, uric acid, and cholesterol molecules. The above materials have to be sensed for a healthier lifestyle for humans and other living organisms. The prescribed review articles give a detailed report on the Electrospun materials for sensor applications.

## 1. Introduction

In recent years, science and technology have tended to develop the sensor research area, which is more sensitive and more versatile than ever before, allowing for even greater potential applications in a wide range of industries. Detection of heavy metal ions or toxic elements in the real sample analysis is much more important, in terms of being directly tied up with the health of living organisms. One of the main advantages of sensors and biosensors is their ability to provide rapid and accurate results, making them highly valuable in time-sensitive situations. Additionally, biosensors are typically portable and easy to use, making them ideal for fieldwork or remote monitoring. As such, biosensors are poised to play an increasingly important role in shaping the future of healthcare, environmental monitoring, and many other areas. One of the most promising areas of biosensor research involves the integration of nanotechnology into biosensor design. By using nanoscale materials and structures, researchers are able to create sensors with unprecedented sensitivity and selectivity. For example, nanowires can be used to create highly sensitive electrical biosensors that can detect even trace amounts of specific molecules. Similarly, nanoparticles can be used to create optical biosensors that are able to detect changes in light absorption or emission in response to the presence of a particular analyte. These advances in nanotechnology are opening up new possibilities for biosensors and biomedical. Since the last decade, the design and development of biosensors have become a top priority for researchers and scientists due to the wide variety of uses for biosensors in areas such as medication delivery, environmental monitoring, water and food quality monitoring, and health care and illness detection. Numerous organizations today are looking for cutting-edge sensors that can react to a range of measurements. An ideal sensor must have specific qualities, including range, drift, calibration, sensitivity, selectivity, linearity, high resolution, reproducibility, repeatability, and response time. The complexity of sensor categorization methods ranges from extremely simple to highly sophisticated [1]. Extreme instances include categorizing information into only three categories (physical, chemical, and biological) and using hierarchical categories with finely divided subcategories in abstracting publications.

Electrochemical sensors are applied in various industrial applications, including healthcare, automotive, aerospace, and environmental monitoring. Due to the increasing demand for smart sensors, researchers and manufacturers are constantly exploring new ways to improve the performance and functionality of these devices. In addition to these categorization methods, there are also different types of sensors that can be used for different applications. For example, some sensors are designed to detect changes in temperature or pressure, while others are used to measure light or sound. Furthermore, advances in technology have led to the development of smart sensors that can communicate with other devices and make decisions based on the data they collect. As organizations continue to seek out the latest sensor technologies, it is important to consider the specific needs and requirements of each application in order to select the most appropriate sensor. There are many sensing techniques that are available in recent research, such as colorimetric, chemo, resistive, and electrochemical sensors. Among the many sensing techniques, electrochemical sensors provide the most accuracy and fastest detection towards the sample analysis. The most difficult aspects of developing biosensors are miniaturizing biosensing devices and improving transducer performance, such as raising sensitivity, cutting down on response time, and improving reproducibility. Moreover, lowering detection limits even to single molecules and accurately capturing biorecognition signals and effectively converting them into electrochemical, electrical, optical, gravimetric, or acoustic signals (the transduction process). These issues can be resolved by combining sensing technology with nanomaterials, which can be zero to three-dimensional, have a high surface-to-volume ratio, excellent conductivities, shock-bearing properties, and color tunability. For example, a biosensor for detecting glucose levels in diabetic patients could be developed using nanomaterials such as gold nanoparticles functionalized with glucose oxidase. The glucose would bind to the glucose oxidase, leading to a change in the electrical conductance of the nanoparticles and thus allowing for detection. The use of nanomaterials not only improves the transducer’s performance but also allows for miniaturization, making it easier to develop portable biosensors for everyday use.

**Figure 1:**
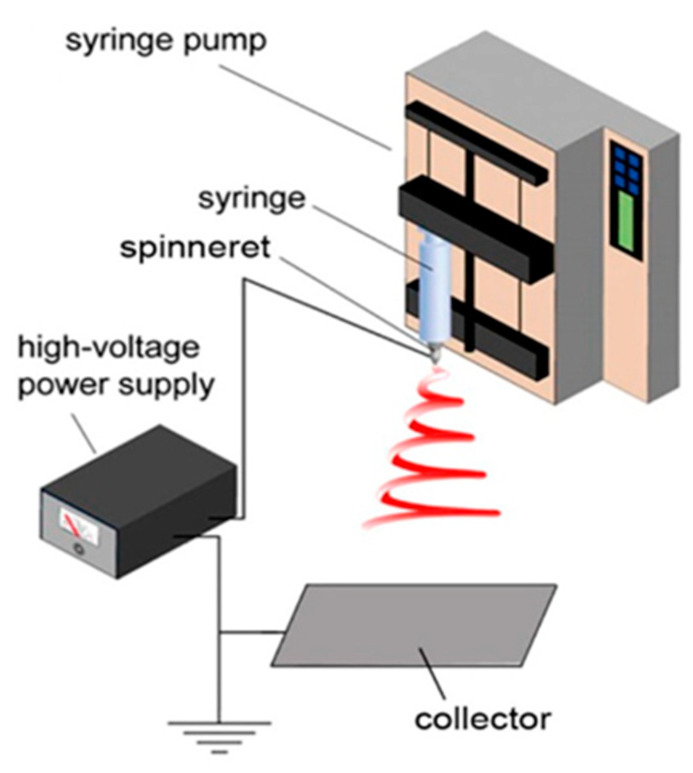
Schematic illustration of a typical electrospinning setup. (Reprinted with permission from Ref. [34]. 2023, American Chemical Society).

Innovative research is particularly interested in nanotechnology, which has sparked the creation of more sensitive, higher-performing sensors. Nanofibers (NFs) have unique features that cannot be found in bulk materials. It was found more than a century ago that a strong electric field may cause ultrathin fibers to be extracted from a viscoelastic fluid. Numerous researchers have rediscovered and improved this process [2,3,4,5,6,7,8,9] known as electrospinning (Figure 1). The amazing characteristics of Electrospun nanofibers, such as their huge portable nature, surface area, flexibility, and porosity, make them an attractive choice for sensor and biosensor applications. They are particularly well suited for large immobilization sites due to their enormous surface area, which leads to greater interaction with analytes. Attention has also been focused on the sensitive identification of compounds of significant physiological importance. Glucose serves as one of the classic examples. To obtain sensitive glucose detection, many kinds of nanofibers have been combined with glucose oxidase or useful nanomaterials (such as semiconducting oxides), offering a reliable platform for glucose monitoring [10,11,12]. Today, a variety of applications and circumstances necessitate the precise detection and quantification of a variety of distinct analytes. The use of biosensors for precise and sensitive detection of proteins, point-of-care testing (POC), the detection of food and environmental toxins, biological warfare agents, illegal narcotics, and the identification of human and animal disease indicators has revolutionized diagnostics [13,14,15,16,17,18]. Due to their extreme specificity for their corresponding antigens, antibodies still play a crucial role in many sensing technologies.

## 2. The Nanofibers and Electrospinning Method

Nanofibers, often referred to as superfine fibers, have a diameter of less than 500 nm and an aspect ratio of at least 100:1. Nanofibers with these characteristics are categorized as one-dimensional (1-D) nanomaterials. Nanofibers can be created using a variety of techniques, including phase separation [19], melt-blown [20], self-assembly [21], solution blow spinning [22], force spinning [23], electrospinning [24], and more. Electrospinning is a process that has been extensively used to manufacture fibers with diameters ranging from nanometers to a few micrometers. It is quite easy to use, it is practical, and it is not very complicated. The ease and low cost of electrospinning as a method for the production of a wide variety of nanofibers in a variety of configurations, including linked fibrous layers, aligned nanofibers, and twisted nanofibrous structures, are perhaps the most significant benefits of this technique. The sources for Electrospun fibers were not just limited to a single component. Polymer composites and hybrid materials, such as polymers with metals, metal oxides, ceramics, and carbon nanotubes, were also included among the sources of Electrospun fibers. Compared to single-component Electrospun nanofibers, two- or multi-component Electrospun nanofibers offer a wider range of compositions, topologies, and functions. This technology has developed significantly as a result of nanofibers and the potential of these fibers in numerous applications. As emphasized previously, the ability to produce nanofibers with controllable dimensions and orientations from a given composition is a direct result of the electrospinning method’s unique properties (capacity for mass production, simplicity of design, and adaptability). Various electrospinning configurations have also been developed as a result of ongoing research into the technique, allowing nanofiber assembly to be tailored to the requirements of specific applications.

Nanofiber can detect changes in the concentration of chemical species, including tiny molecules (such as glucose or water), biomolecules (such as enzymes or proteins), and even gaseous species. For instance, 30 wt.% LiCl-doped porous TiO_2_ nanofibers demonstrated good sensitivity to variations in humidity [25]. The creation of effective transducer nanomaterials for gas sensing has enormous hurdles since they must simultaneously exhibit good selectivity, low power consumption, a quick response or recovery rate, minimal humidity dependency, and a low detection limit [26]. Electrospun nanofibers can also be used to create extremely sensitive optical sensors. GaN nanofibers, for instance, were created via electrospinning [27]. A GaN nanofiber’s conductance rose by 830 times when exposed to UV radiation, more than 10-fold over a single-crystal GaN nanowire’s (about 78 times) conductance when formed via chemical vapor deposition. The polycrystalline structure and rough surface of the Electrospun GaN nanofibers, which produced more photogenerated carriers than a smooth GaN nanowire, were said to be the causes of their high sensitivity.

Nowadays, nanofibers with a high surface area-to-volume ratios and high porosity ratios have been the subject of extensive research in a broad variety of applications. The combination of ultrafine diameter and porosity in porous nanofibers represents an emerging class of nanoporous materials with the maximum imaginable specific surface area, high pore volume, and extreme adsorption capacity, which could result in enhancements to sensor applications. Not only for sensors applications but also for tissue engineering, drug delivery, adsorption and separation materials, catalysts, supercapacitors, energy storage, superhydrophobic materials, batteries, conductors, fuel cells, dye-sensitized solar cells, and filtration. Recently, the fabrication of these nanofibers has been appreciated by a number of groups.

### 2.1. The Development of Electrospinning

During the 1990s, the electrospinning industry experienced explosive growth as a direct result of the development of nanotechnology. Boys conducted the first experiments with electrical spinning in 1887 with the goal of producing the finest thread [28], but researchers were not particularly interested in this method at the time. In the year 1902, Cooley and Morton were granted a patent for the electrospinning procedure and the associated equipment. In the 1960s, Taylor mathematically derived the conical geometry for fluid droplets when an electric field is present. This geometry is known as the “Taylor cone” [29]. It has been demonstrated that the electrospinning process is an efficient method for the production of fibers that possess exceptional qualities. Researchers have found that the diameter control, morphology, and alignment of the fibers can be changed by adjusting the parameters of the electrospinning setup [30,31]. These parameters include the voltage, the distance between the spinneret and the collector, and the flow rate of the polymer solution. Electrospinning has become a popular method for the fabrication of materials for a wide variety of applications, including tissue engineering, drug delivery, and energy storage, as a result of the high level of control it offers. Electrospinning is another method that researchers have investigated in recent years for the purpose of producing fibers from a variety of materials.

The setup substantially consists of a spinneret, collector, and high voltage power force. An optimum voltage (in kV) is applied between the spinneret and a collector; both are electrically conducting and separated at an optimum distance. Electrospinning of accouterments involves the application of a strong electric field to a drop of fluid, melt, or mix result. Figure 1 displays a diagrammatic representation of an electrospinning setup. A controlled amount of the polymer solution or melt is dispensed through the tiny needle aperture while being subjected to the electrostatic force generated by the high-voltage source. The polymer melt gets discharged due to the accumulation of charge at the spinneret orifice. After that, the polymer melt that has been discharged will be subjected to the electric field. When the high voltage is gradually raised, polymer droplets elongate into a conical form known as the Taylor cone [32,33] that is subsequently stretched to produce a jet. At first, the jet expands along a straight path; however, when it reaches a certain point, bending instabilities cause it to begin violently whirling. The oppositely charged collector, or the grounded collector, is where the drawn polymer thread is directed to go. As the jet is compressed into smaller dimensions, it immediately solidifies, causing solid fiber(s) to deposit on the grounded collector, where the charged polymer fiber is the only thing left on the collector mat after the residual solvent has evaporated [34].

### 2.2. The Nanofiber Formation by Electrospinning Method

In the process of electrospinning, the liquid is typically pumped into the spinneret at a predictable and consistent rate using a syringe. Within the liquid, positive and negative charges will begin to separate as a result of the potential difference that exists between the spinneret and the collector. Surplus charges are produced as a result of the movement of charges toward the surface of the droplet that have the same sign as the polarity of the spinneret. The accumulation of additional charges on the surface of a droplet leads to a higher density of surface charges when the voltage is gradually increased. Surface tension encourages a spherical shape in order to reduce the total surface free energy of the droplet. This is in contrast to electrostatic repulsion, which attempts to distort the shape of the droplet. Because of this, its surface area will be increased, so the repulsion will be reduced [35]. When a droplet is electrified, the electrostatic attraction between surface charges of the same sign causes the droplet to become deformed into a Taylor cone. When the electrostatic force is strong enough, it will overcome the surface tension of the droplet, which will result in the release of a finely charged jet from the bottom of the Taylor cone [36]. The electrostatic field then causes a sequence of whipping instabilities in the fine-charged jet, which causes it to speed into thin fibers and quickly evaporate the solvent [37]. The elongation process hardens the jet into fibers, which are brought on either by the solvent evaporating or the melt cooling. The elongation of the charged jet can persist for a longer duration to produce fibers with a narrower diameter when the solidification process is sluggish.

The deposition of fibers on a grounded collector is the last stage of an electrospinning procedure [38]. The bending instability stage in which the fibers are deposited has a significant impact on the morphologies of the fibers. The fibers in the loop area of the initial bending instability can easily be collected as a nonwoven mat on a stationary or moving collector. The coils can be gathered as fibers with a straight or wavy morphology or even coils with many turns. However, the fibers in the tiny, coiled area of the second and third bending instabilities may take on a complicated structure. Most of the charges that are deposited on the fibers afterward are immediately discharged through the grounded collector [39,40]. The size and shape of the resulting fibers can be controlled by adjusting the parameters of the electrospinning process.

### 2.3. Electrospinning Parameters and Factors Affecting the Morphology of Nanofibers

Based on a balance between the electrospinning parameters and the solution properties, nanofibers can be created via electrospinning. It is necessary to adjust the solution’s viscosity, dielectric properties, and surface tension in order to produce homogeneous nanofibers by balancing these factors with the electrostatic forces produced by the chosen feed rate, collector distance, and high-voltage tension. Three key variables that affect the ultimate fiber shape and diameter are setup parameters, polymer solution parameters, and environmental variables.

1. Setup parameters include the feeding rate, diameter, and shape of the spinneret, separation between the collector and the spinneret tip, and collector’s shape.
2. Polymer solution parameters include additives, solvents, polymers, and properties.
3. Environmental variables include temperature, relative humidity, and gas velocity.

As a result, no set of universal electrospinning settings can be used with all polymers. Electrospinning may create a large variety of polymer fibers with various morphologies/structures and diameters ranging from a few nanometers to several micrometers, but by carefully adjusting the parameters listed above. The gathered fibers have an increased modular strength and a nano/microscale diameter. They are excellent candidates for biological application since they also resemble the ECM [41]. The morphological features of the synthesized Electrospun fibers frequently need to be adjusted to construct patterned nanostructures with the aim of enhancing the performance metrics of bio-receptors such as response time, stability, and sensitivity. This is frequently accomplished by adding chemicals before, during, or after the electrospinning process that are immobilized inside or on the surface of the nanofibers. Inorganic precursor solutions (metal salts or metal alkoxides) typically have unsuitable viscosities and high hydrolysis rates that hinder the creation of stable jets during the spinning technique. Since the polymer should serve as a structural matrix, two methods have been suggested to address this drawback. One is to use metallic precursors, such as metal salts, dispersed in a polymeric solution for attaining proper viscosity [42] or by using metallic alkoxides that are subjected to polymerization to form a sol-gel, with a catalyst to control the hydrolysis rate and then the combination with a polymeric solution for attaining suitable viscosity [43]. Typically, during the electrospinning process, both stable and unstable electrospinning jets can be seen. The “Plateau-Rayleigh instability” causes the jet to disperse into droplets if the supplied external electrostatic force is less than the critical voltage [44,45,46]. The steady electrospinning jet can be produced if the supplied external electrostatic force exceeds the critical voltage within a specific range. Two, three, or even four electro-rotating jets can be created from a single droplet when an external electrostatic force that is substantially greater than the surface tension is applied [36]. The electrostatic repulsion between the surface charges produces a lateral force (F_R_) that forces the jet to bend. Due to bending instabilities, the jet first expands in a straight path before undergoing ferocious whipping movements. An electrically charged jet may experience three different forms of instabilities in the far-field area. The first kind, also known as Rayleigh instability, is axisymmetric and may cause the jet to fragment into droplets [36]. Surface tension dominates it, and a strong electric field can inhibit it. When compared to the first kind, the second type has a higher electric field and is likewise axisymmetric. Non-axisymmetric instability is the third category, commonly referred to as whipping or bending instability. It states that the aerodynamic instability and the “lateral electrostatic force” in a radial direction relative to the jet, which is caused by the electrostatic repulsion between surface charges in a high electric field, are what cause long wave perturbations to the jet. The advantages of the electrospinning nanofiber in electrochemical sensor as follows: (i) the nanofiber material preparation is much easy and cost effective, (ii) the rate of sensitivity and selectivity is much higher than other methods, (iii) the detection level is very lower in concentrations, (iv) high recycle stability, and (iv) friendly to the environmental surroundings. These above concerns may be also applicable to the energy and electrochemical industrial application.

Despite the fact that Electrospun nanofibers have been employed effectively in many different applications, they suffer from various issues including safe fabrication and scale-up production [45]. Most of the natural polymer or biopolymers are insoluble or poorly soluble in aqueous or organic solvents. Processability and electrospinning of such polymers in to nanofiber form is challenging. Moreover, the production of nanofibers with multifunctional and porous nature often requires toxic organic solvents and high electric field which limits the electrospinning technique. To overcome these issues, various new approaches such as water-borne emulsion electrospinning have been developed. In addition, mass production of nanofibers from water soluble polymer such as PEO, PVA and PVP are developed [41,42,43,44,45,46]. Overall, although the electrospinning technique has disadvantages, for most of the cases it is worked well in developing highly efficient material for various applications such as biomedical, catalysis, water filtration, energy and separator. In particularly, the electrospun nanofibers played a crucial role as an electrochemical sensor material. Hence, the present review mainly focuses on electrochemical sensing application of Electrospun nanofiber materials. 

## 3. Chemical Sensors

Excess heavy metal ions and some toxic chemicals are major threats for normal living organisms. On the other hand, some bio-based molecules, such as glucose, uric acid, and cholesterol, have to be controlled at a normal level. So, the detection of hazardous chemicals and bio-based molecules is very important for the global environment. There are many types of diagnostic methods available to detect the above ions and molecules. Among these, electrochemical sensors are more effective in terms of easy handling and fast response. Electrochemical devices are those that can either convert chemical reactions into electrical energy or electrical energy into a chemical reaction. Examples of galvanic cells are batteries, fuel cells, supercapacitors, solar cells, and sensors. Electrolytic cells are used to decompose chemical compounds through the process of electrolysis. Nano-structuring the electrodes can increase the contact area between the various parts of the electrochemical device, typically the electrodes and electrolytes [47]. Sensors can be optical, electrochemical, electrical, mass sensitive, thermometric, and magnetic. The receptor part, which is in charge of the recognition of the analyte, can have its foundation in either physical or chemical principles. If the recognition process is based on a biochemical reaction, the sensor is called a biosensor [48,49].

The chemical sensor involves the detection of hazardous molecules from the specified samples. The hazardous chemicals have to be identified before real-time usage. The detection of chemicals on various samples is much more important in terms of quantifying the level of the chemical present in the real samples. Here are some examples of hazardous chemical detection using various approaches.

### 3.1. Volatile Organic Compound (VOC) Sensor

The term VOC impels as the hazardous gases emitted from the particular solids or liquids. The reason for the VOC is owing to the lower boiling point, photochemical reactions, atmospheric pressure, etc. The case VOC is more sensitive and needs more attention than other chemical compounds, because it directly attacks the human system by breathing hazardous gas. The common VOC compounds that persist in the laboratory are acetone, ethanol, and ammonia. The detection of the above chemicals by Electrospun nanofiber composites are as follows.

#### 3.1.1. Acetone Sensor

**Figure 2:**
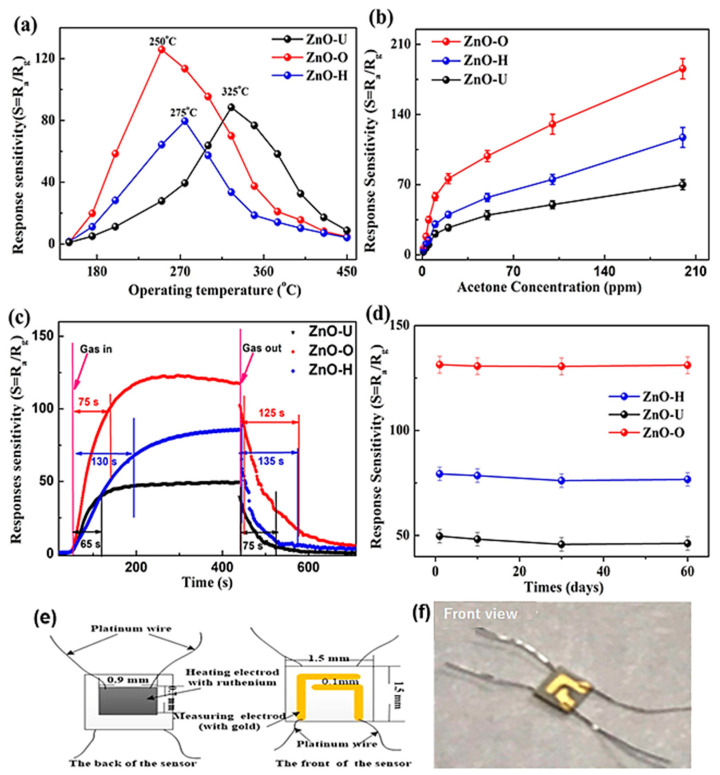
The sensitivity response (**a**) of the materials towards 100 ppm acetone and various concentration at 250 °C (**b**); recovery time of the materials on 100 ppm acetone (**c**); recycle test of the materials for acetone sensing in 60 days at 250 °C (**d**); schematic illustration of the depletion layer thickness and potential barriers of ZnO nanofibers (**e**,**f**).

Acetone is the most commonly used solvent in chemical laboratories; it has a lower boiling point. So, this can be volatile and spread to the environment; an excess of acetone is highly dangerous to living organisms and humans’ health as well. So, it is important to detect the level of acetone in the atmosphere. Here are some various acetone sensor materials, as follows:

The oxygen plasma-treated ZnO nanofiber was used to detect the acetone; first, the ZnO nanowire was prepared by electrospinning, followed by the oxygen plasma treatment on the surface of the ZnO nanowire (Figure 2). The prepared material was characterized using various analyses, such as P-XRD, FE-SEM, XPS, and BET analysis. The sensing properties of the materials were studied by a static measurement device. The surface treatment increases as the pores and surface area of the ZnO nanowire increase. The increase in surface area leads to an enhancement of the adsorption energy of the ZnO nanowire surface area. The dopant of oxygen molecules on the surface of the ZnO nanowire also enhances the charge transfer nature of the materials, which makes it further feasible to attach the acetone molecule to the surface of the material. Finally, the gas sensing property of the oxygen plasma-treated ZnO nanowire is twice as good as that of the untreated ZnO nanowire [50].

**Figure 3:**
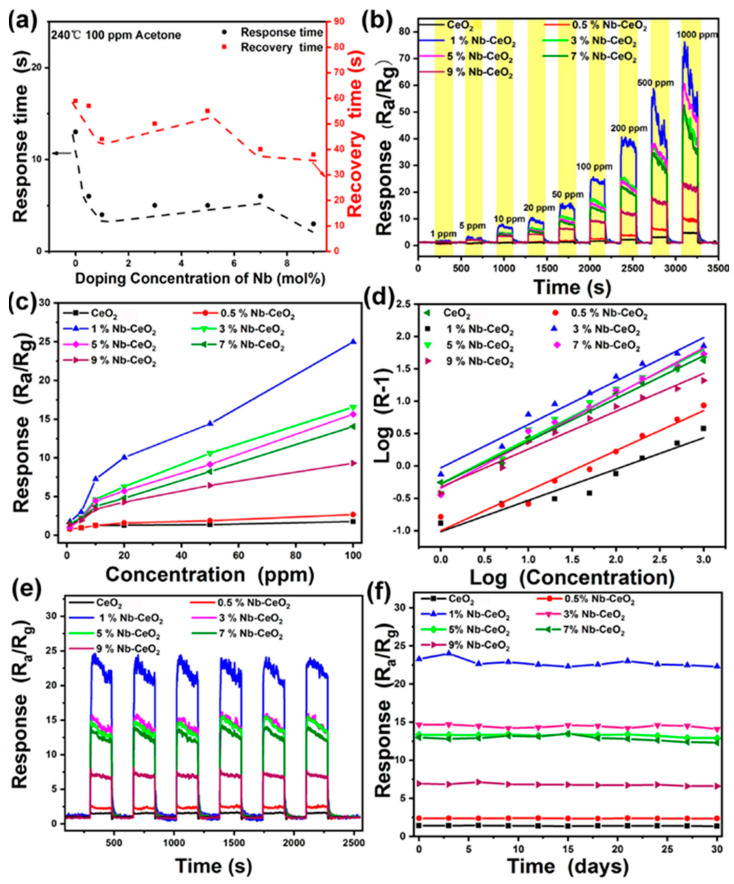
The recovery time of the material and different concentration (**a**) to 100 ppm acetone; (**b**–**d**) response for the different time and concentration of acetone (1–1000 ppm); recycle and stability test of the prepared materials (**e**,**f**).

Metal oxide-doped nanofibers have recently taken on an important role in sensors and technology due to their high selectivity and sensitivity toward the analyte materials (Figure 3). The niobium-doped cerium oxide material has been proposed for the humidity-independent acetone sensing application. The various percentages (%) of metal oxide composite materials were prepared by the efficient electrospinning method. The materials were characterized by routine, sophisticated analysis. Among the various concentrations, the 1% of Nb-doped CeO_2_ shows 13.37 times higher values than the undoped metal oxide. The reason behind the enhancement in the sensing mechanism is that the higher oxygen vacancy leads to enhanced sensing behavior of the composite material [51].

**Figure 4:**
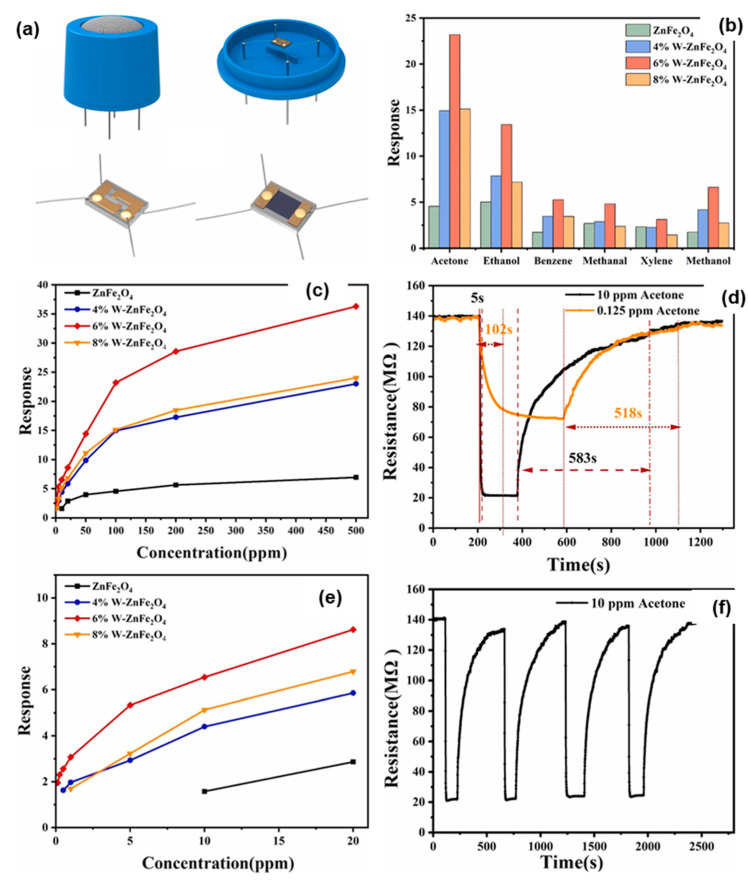
The schematic of the gas sensor (**a**), the front and back of the Al_2_O_3_ substrate; gas responses of sensors based on W-doped ZnFe_2_O_4_ to 100 ppm of various target gases at optimum operating temperature (**b**); response on doped and undoped materials for acetone concentration (**c**); the sensing results of 10 ppm and 0.125 ppm acetone (**d**,**e**) (dotted redline in (**d**) indicates response time difference between two different acetone concentrations); four periods of the response curve towards 10 ppm acetone at 200 °C (**f**).

Tri metal oxide-based nanowires are much more sensitive toward acetone detection. These types of materials can detect even lower concentrations of acetone also. The W-doped ZnFe_2_O_4_ was prepared from the electrospinning process method and analyzed for acetone detection (Figure 4). The morphology and the crystalline phase of the materials were varied with respect to the concentration of the W; the olive-shaped nanoparticles were obtained on the surface of the nanowire. The 6% mol W-doped ZnFe_2_O_4_ materials achieved the highest response towards the detection of acetone as 0.125 ppm acetone vapor at 200 °C. The W-doping on the surface of the nanowire is five times higher than the un-doping nanowire [52].

#### 3.1.2. Ethanol Sensor

**Figure 5:**
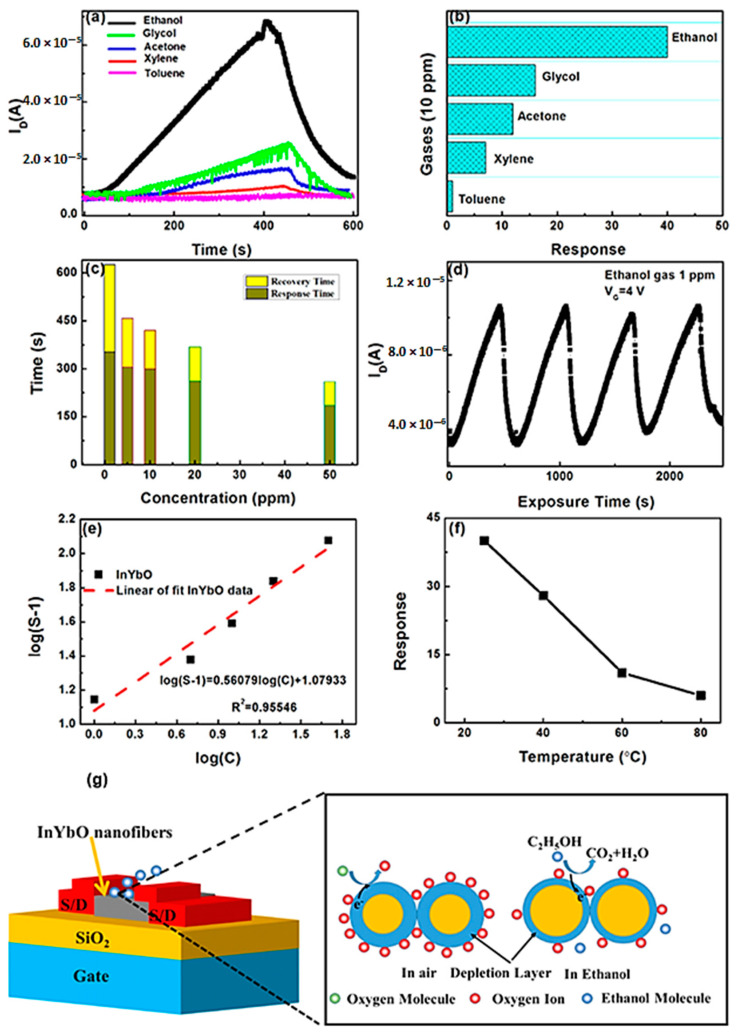
Dynamic response result of FET nanofiber to various gas and ethanol 10 ppm (**a**,**b**); response and recovery of the materials on 1–50 ppm concentration (**c**); sensing response of InYb4%O for four cycles (**d**) and linear relationship (**e**); response of InYb4%O nanofiber FET gas sensors to 10 ppm ethanol gas as a function of the working temperatures (**f**); schematic diagram of the ethanol gas-sensing mechanism of the material (**g**).

Ytterbium doped indium oxide materials are prepared as a nanowire and used for the detection of ethanol in field effect transistor (FET) devices (Figure 5). The materials were prepared in a simple and fast manner, a concentration of 4 mol % ytterbium-doped on the In_2_O_3_ and formed as an InYbO nanofiber; these device shows better electrical performance with an ideal charge transporting nature. Besides that, the prepared nanowires detect the ethanol in lower concentrations (40–10 ppm) with high response. Also, these materials can detect 1 ppm of ethanol with sensitivity and selectivity [53].

**Figure 6:**
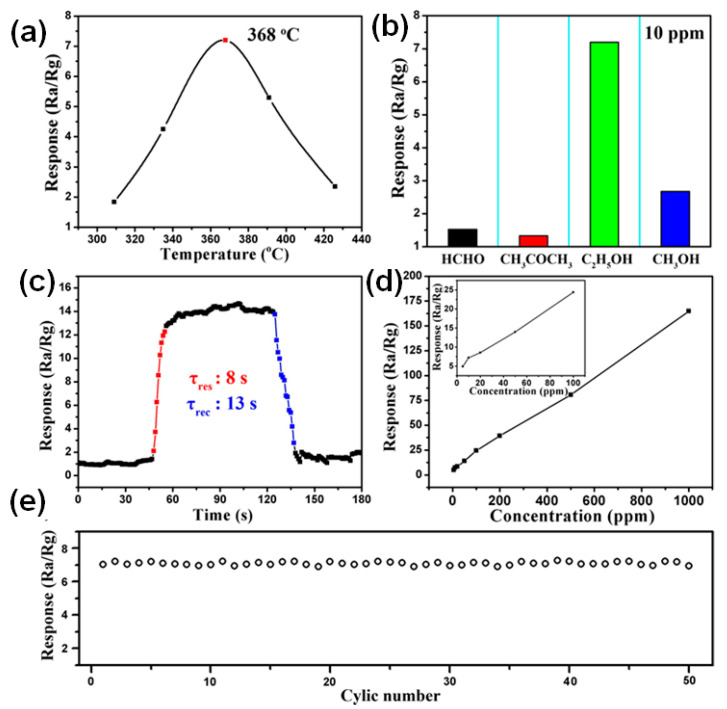
The operating temperature response on 10 ppm ethanol (**a**) and selectivity of sensor (**b**). Dynamic response of sensor to 50 ppm ethanol (**c**); various concentration (5–1000 ppm) result (**d**), response and recycle stability of the material (**e**).

The [PVP/Ti(SO_4_)_2_]//[PVP/SnCl_4_] and (b) TiO_2_//SnO_2_ Janus NFs based nanofiber was used to detect ethanol in the level of 10 ppm. The nanofiber was prepared from a parallel method using electrospinning technique. The sensing results against ethanol shows good selectivity and fast response as well. The obtained results were depicted in Figure 6. The possible mechanism of ethanol is as follows [54].

$$\begin{array}{l} O_{2,(\mathrm{ads})}↔O_{2,(\mathrm{ads})}^{-}↔O_{\left(\mathrm{ads}\right)}^{-}↔O_{\left(\mathrm{ads}\right)}^{2-}\\ C_{2}H_{5}{\mathrm{OH}+6O}_{{}_{\left(\mathrm{ads}\right)}}^{-}\to {2\mathrm{CO}}_{2}{+3H}_{2}{O+6e}^{-} \end{array}$$

**Figure 7:**
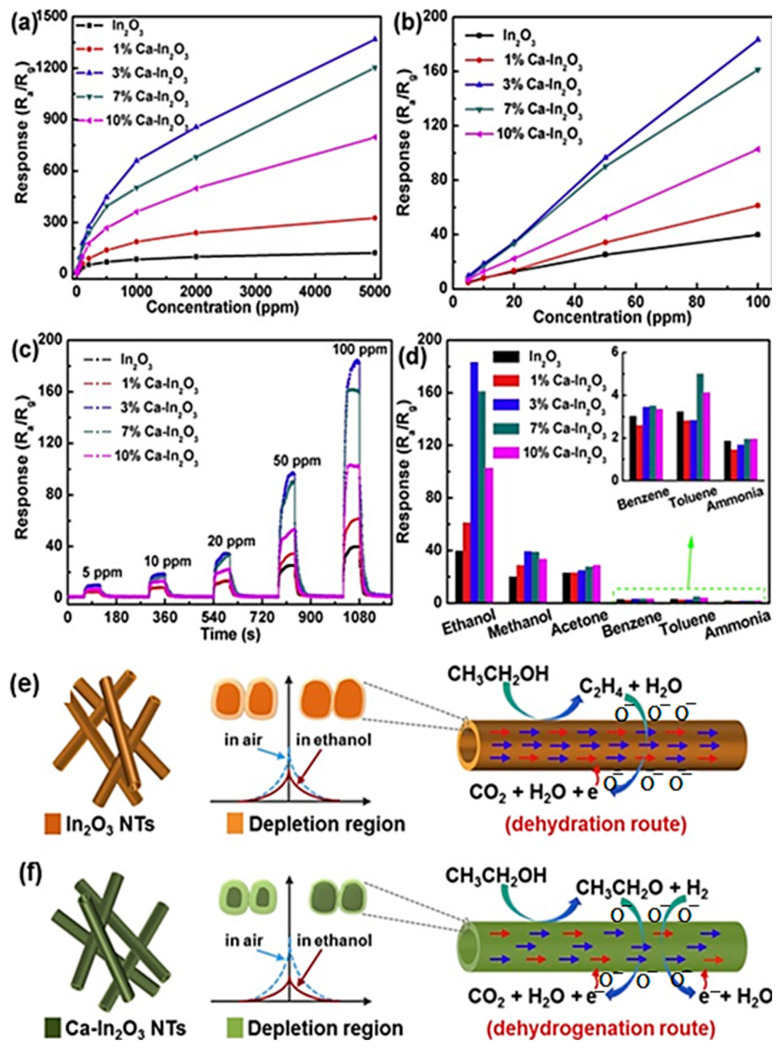
The ethanol gas sensing results of fiber composites materials (**a**–**d**); the schematic representation on sensing mechanism of doped and undoped materials (**e**,**f**).

The Ca-doped In_2_O_3_ nanotubes were prepared by the electrospinning method and the amount of calcium was varied from 7 to 10 mol % to form CaIn_2_O_4_ nanotubes (Figure 7). The materials prepared in terms of selective and sensitive detection of ethanol, and the dopant materials have affected the crystal structure of the In_2_O_3_. The results suggest that as the dopant of calcium enters into the lattice of the In_2_O_3_, which enhances the vacancy of the oxygen, the particle size of the materials may enhance with respect to the doping of calcium. The prepared nanotubes show the highest response towards the ethanol of 183.3 at 100 ppm, with an outstanding sensing of ethanol at 240 °C. The doped nanotube materials show enhanced sensing nature than undoped material, and the excellent detection of ethanol is due to the incorporation of the calcium ions into the In_2_O_3_ [55].

#### 3.1.3. Ammonia Sensor

**Figure 8:**
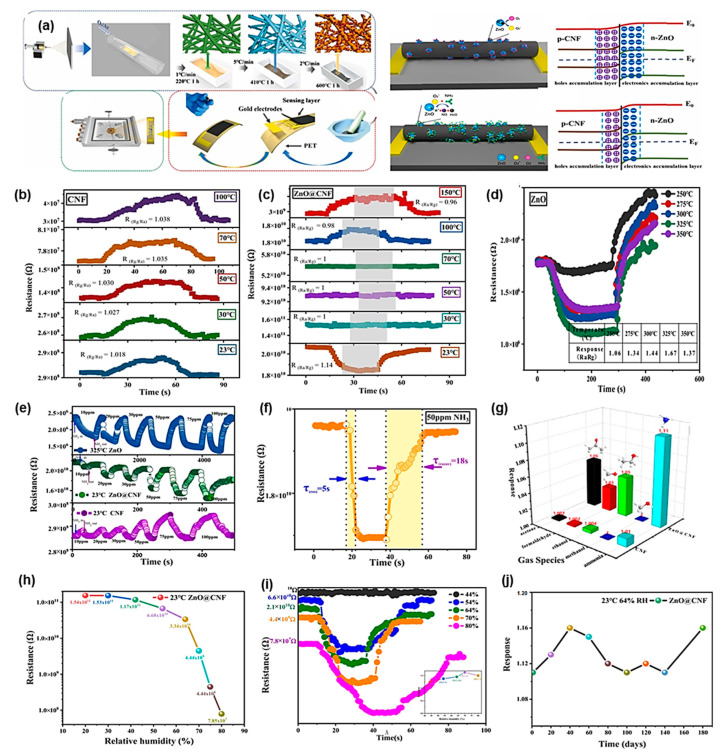
Schematic illustration of electrospinning and device fabrication of the composite (**a**); sensing analysis on various temperature, concentration and various parameters (**b**–**i**); stability test of the ZnO@CNF material on 180 days and (**j**) Resistance curve of ZnO@CNF vs relative humidity at 23 °C.

The ammonia is easily dispersible and the most hazardous gas in the laboratory. The leakage of pipelines or some by-product ammonia exposed the outside. The detection of ammonia is very important to control the leakage of the ammonia. The ZnO nanoparticles have modified on the surface of the carbon nanofiber by electrospinning method followed by the peroxidation, oxidation, and calcination (Figure 8). The advantage of these materials includes highly flexible and response to the ammonia. The peroxidation involves cyclization, dehydration, and oxidation process; the calcination process involves the compression of carbon fiber diameter. The prepared composite materials are good enough for ammonia detection and much better than the starting materials of CNF and ZnO. The merits of the composite towards ammonia detection as highly flexible and sensing is even better in room temperature also [56].

**Figure 9:**
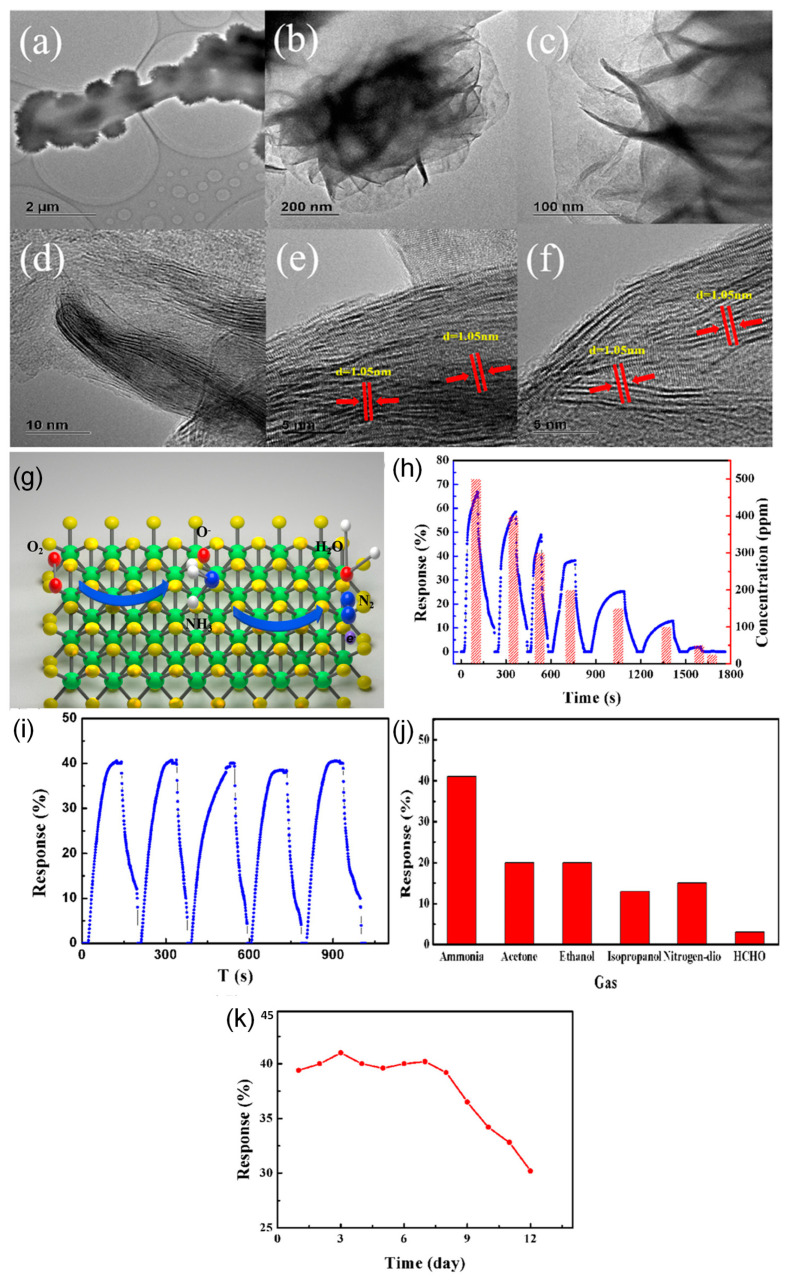
The HR-TEM images of MoS_2_ nano chain material (**a**–**f**); schematic mechanism of ammonia sensing on the surface (**g**); results on sensing of ammonia at 25-500 ppm (**h**), recyclability (**i**), selectivity (**j**), long-term stability (**k**).

The MoS_2_ and GO based nanochain material was prepared by electrospinning followed by hydrothermal method (Figure 9). The morphological analysis reveals the materials show three-dimensional hydrangea-like MoS_2_ nanospheres structure in FE-SEM and the single layer structure was confirmed by the HR-TEM analysis. The Raman and P-XRD spectra confirm the formation of the material, while the XPS confirms the elemental composition of the materials. The sensing of ammonia was tested in the range of 25–500 ppm; the detection response increases with respect to the concentration of the ammonia. The nanochain formation is the reason for the high sensitivity of the molecules towards the ammonia, with the maximum results obtained at 71% at 500 ppm of ammonia [57].

### 3.2. Carbon Dioxide Sensor

**Figure 10:**
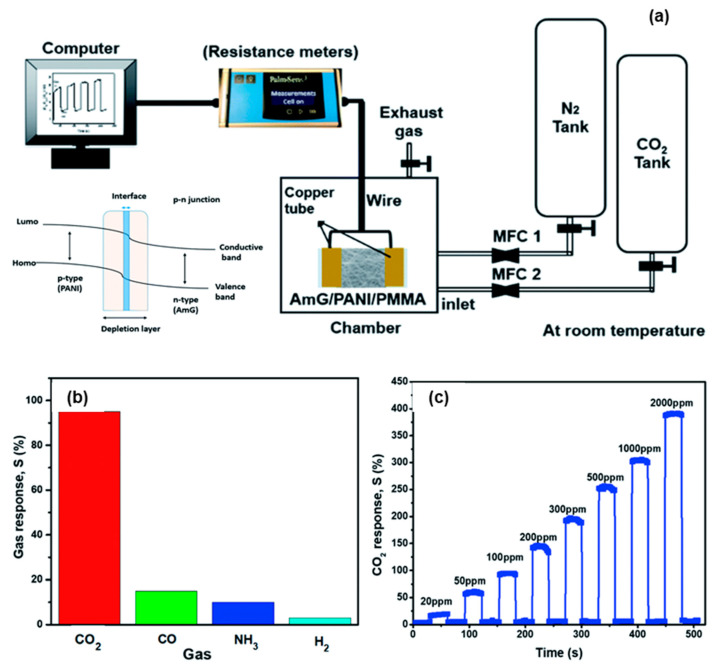
Instrumentation setup of CO_2_ sensor (**a**); the selectivity of the AmG/PANI nanofiber composite gas sensor to various gases (**b**); the response of gas sensor to different CO_2_ concentrations (**c**).

Graphene and poly aniline composite materials were used to detect the carbon dioxide gas in room temperature. The composite material was prepared by routine electrospinning method to obtain a nanofiber structure (Figure 10). The two types of nanocomposites as PMMA/PANI and amino functionalized graphene/PANI were compared to the detection of CO_2_. Among these, the AMG/PANI shows the better electrical response while CO_2_ passing. The AMG/PANI shows the faster response and high sensitivity towards the CO_2_ detection [58].

**Figure 11:**
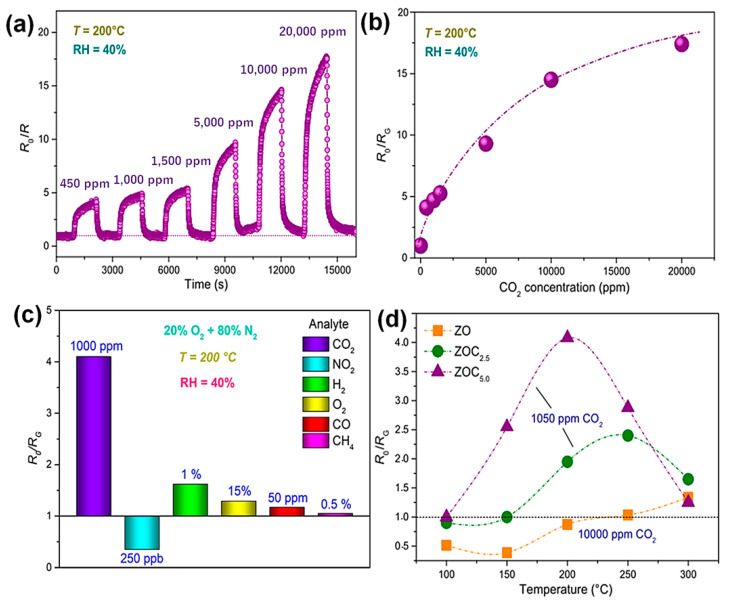
The dynamic response of the materials on different concentration (**a**) of CO_2_, calibration curve (**b**), different gas (**c**), and different temperature (**d**) results of the Ca-doped fiber composite material.

The calcium-doped zinc oxide nanofiber materials were prepared by electro spinning method and used to detect the CO_2_ molecule (Figure 11). The advantages of this nanofiber material are low cost and high response towards the sensing of CO_2_. The different ratio of calcium and zinc as (1:40 and 1:20) was fabricated through the electrospinning method. The formation of the materials is based on the interconnected grains of oxide with the hexagonal wurtzite structure of zincite. The ratio of 1: 20 shows better CO_2_ detection than 1: 40 ratios. The synergetic effect between the layer is the reason for the fast and sensitive detection of the CO_2_ molecule [59].

### 3.3. Hydrogen Peroxide (H_2_O_2_) Sensor

Hydrogen peroxide is more hazardous chemical towards the environmental and living organisms. The qualitative and quantitative analysis of H_2_O_2_ is very important to prevent the dangerous issues. The H_2_O_2_ detection by the materials prepared from electrospinning method is much sensitive and faster response. The preparation of the Electrospun metal oxide materials are much easier and effective than other types of preparations. The advantages over other materials include fiber structure and uniform dissociation of the active material throughout the active matrix as well.

**Figure 12:**
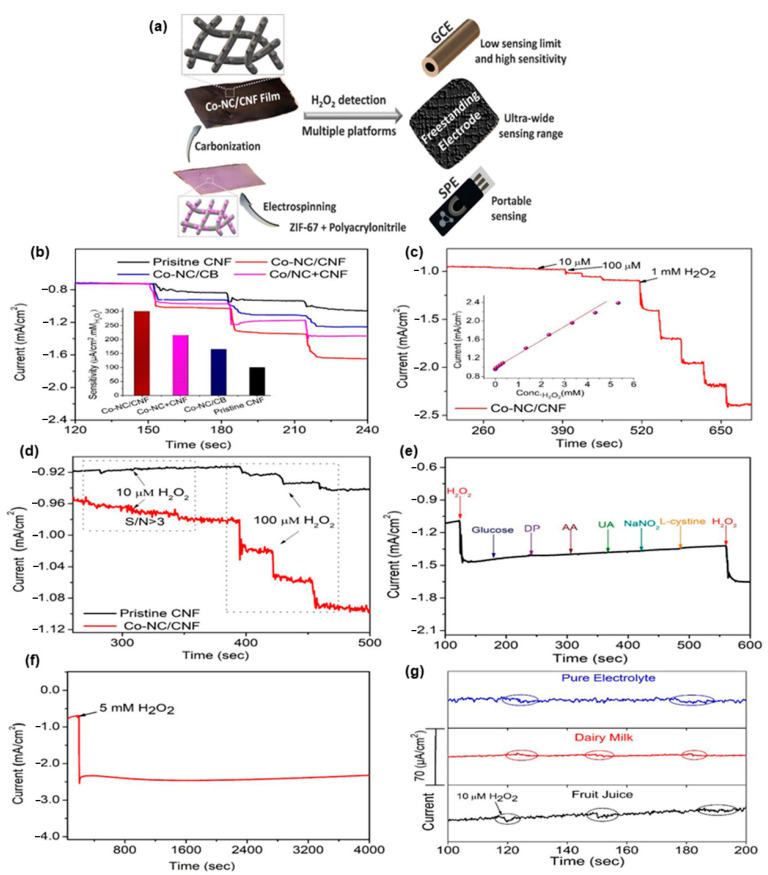
Schematic representation (**a**) of H_2_O_2_ sensor by Co-NC/CNF film; the sensitivity (**b**) and *i*-*t* cure (**c**) of Co-NC/CNF on different H_2_O_2_ concentration; (**d**) current responses to probe the detection limit of Co-NC/CNF and pristine CNFs; (**e**) the selectivity, stability (**f**), and real time food sample (**g**) analysis result of the material.

The ZIF-67 (zeolite imidazole frame) is a typical MOF material, which is prepared from the 2-methylimidazole and cobalt. These MOFs form as tetrahedral coordinated divalent Co^2+^ metal ions with the 2-methylimidazole. The uniform crystalline of formation network of the molecule leads to possess the high surface area of the material. The unique property of the ZIF-67 tends to be used in various applications, such as sensors, molecular separations energy, and environmental technology. The composition of ZIF-67 and polyacrylonitrile were prepared from Electrospun technique. The nanofibers further carbonized to get the cobalt impregnated carbon nanofiber material. The cobalt substituted carbon nanofiber was then fabricated as electrode material to detect the H_2_O_2_. The figures suggest the electrochemical analysis of pristine CNF and cobalt modified CNF materials (Figure 12). The obtained results suggest the cobalt functionalized CNF possess the efficient sensor property in nature. The material gives better efficiency even in lower concentration such as 5 mM H_2_O_2_ also. The fabricated cobalt modified CNF device was also tried with real time analysis, in terms of milk and juice products. The obtained results show the device is good for the detection of 10 Mm H_2_O_2_ and are also optimum to applicable in real time application [60].

### 3.4. Nitric Oxide (NO) Sensor

**Figure 13:**
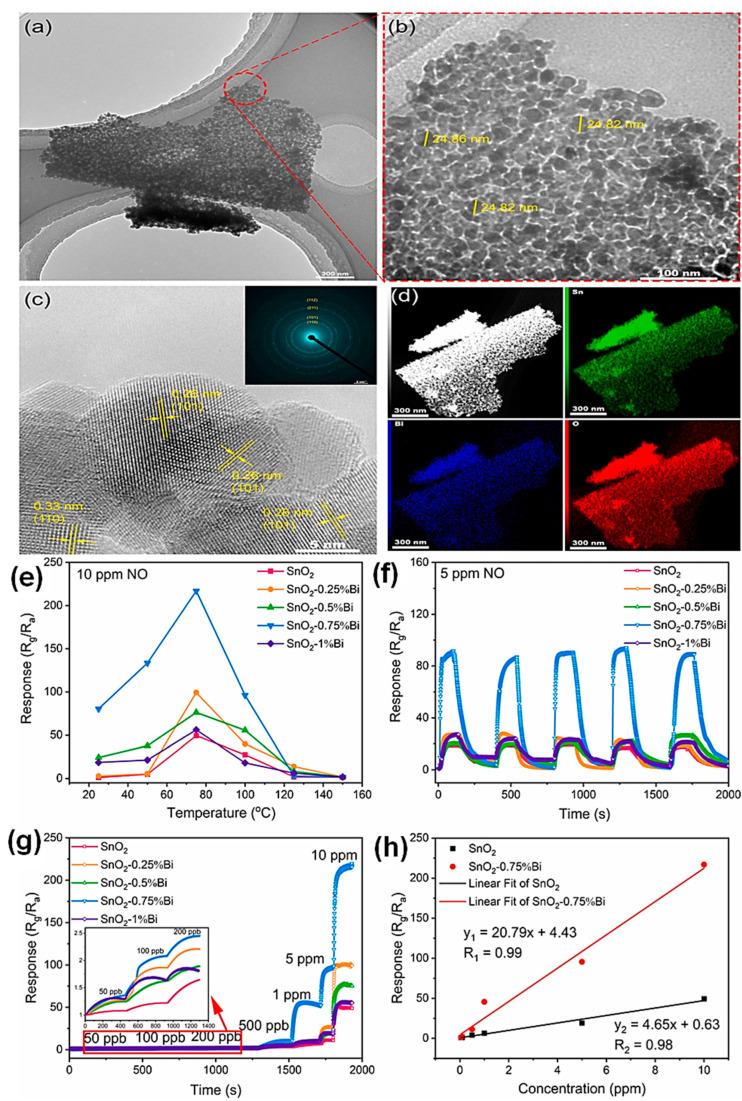
(**a**,**b**) TEM images, (**c**) HR-TEM (inset is the SAED pattern), and (**d**) element mapping images of SnO_2_-0.75%Bi; (**e**) the response of all samples to 10 ppm NO under various operating temperatures; (**f**) the repeatability of the pure SnO_2_ and different concentrations of Bi-doped SnO_2_ upon a five-cycles exposing to 5 ppm NO at 75 °C; (**g**) the dynamic curves of pure SnO_2_ and different concentrations of Bi-doped SnO_2_ sensors to NO with the concentration of 50 ppb-10 ppm at 75 °C. The inset is a 50–200 ppb enlargement; (**h**) gas responses of pure SnO_2_ and SnO_2_-0.75% Bi samples as a function of NO concentration at 75 °C.

Nitric oxide is a highly hazardous colorless gas; this gas occurs as a radical ion with heteronuclear diatomic molecule. The nitric oxide obtained a intermediate molecule with highly unstable condition. The leakage of nitric oxide leads to dangerous accidents, so it is important to detect the nitric oxide in laboratory conditions. Bismuth-doped SnO_2_ porous nanostructures were used to detect the nitric oxide in a selective and sensitive approach. The ultra-thin nano sheet with porous structured bismuth SnO_2_ composites materials was prepared using electrospinning method. The prepared material was shown to have better efficiency concentration detection of 10–270 ppm NO at the temperature of 70 °C and the lower detection was obtained at 50 ppb. The stability and the recycle uses were also good enough for the further usage of this material towards the NO detection in the real time analysis (Figure 13) [61].

## 4. Biosensors

**Figure 14:**
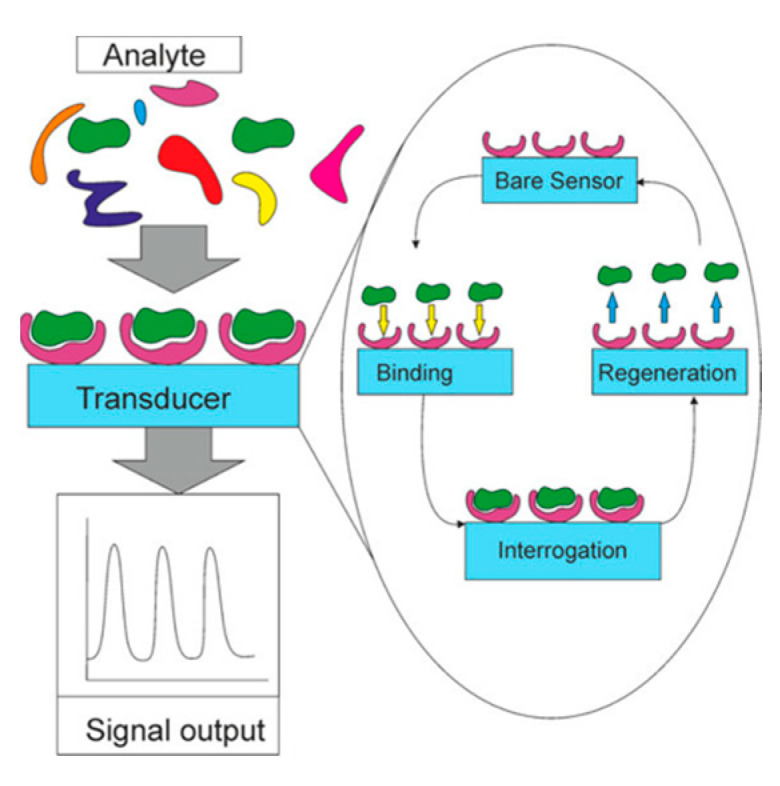
Diagram depicting the operation of biosensors (**left**) and their regeneration (**right**). Regeneration is carried out following analyte binding and interrogation in order to restore the sensor and bioreceptor to their initial configuration (Reprinted with permission from Ref. [69]. 2023, American Chemical Society”.) [69].

Bio molecules such as glucose, starch, sucrose, fructose, and uric acid are facing difficult to detect the materials from the sources, due to their complicated structure. The biosensor involves the finding of biological molecules by using respective sensor molecules. A biosensor is an integrated receptor–transducer device that can convert a biological reaction into an electrical signal (Figure 14). The term “biosensor” refers to the instrument that is utilized for the purpose of detecting biological substances such as biomolecules, transducers, or bacteria. The operation of biosensors is comprised of three essential components: first, a portion that aids in the discovery of an analyte and the production of a signal; second, a signal transducer; and third, a reader device [62]. Utilizing biosensors, it is possible to measure the activity of body-found enzymes and determine the viability of stem cells. Enzymatic and nonenzymatic biosensors are the two primary classifications of this type of sensor. Enzymatic biosensors have the potential to increase sensitivity due to their many advantageous features, such as their high selectivity, high performance, and high catalytic activity. In spite of the virtues that have been presented, contemporary research has frequently concentrated on enzyme-free biosensors. Because of the high cost of enzymes, thermal and chemical instability, and the process of enzyme immobilization, as well as the reliance of sensor sensitivity on temperature, pH, and humidity [63,64]. For instance, biosensors may struggle to differentiate between structurally similar molecules such as glucose and fructose, which both have six carbon atoms arranged in a similar way. This can lead to inaccurate measurements and hinder the performance of biosensing devices. In recent years, there has been a rapid development of biosensors and bioelectronic devices that are capable of monitoring human health. However, the primary obstacles that stand in the way of the development of biosensors at this time are poor stability and a limited lifespan. After the invention of nanotechnology, new doors of opportunity opened for the development of biosensors due to the fact that it has improved specificity, detection time, sensitivity, and cost while simultaneously lowering costs. The nanostructure of the biosensor can range in size from one to one hundred nanometers (nm), and its shape can also change depending on the type of system it is made of (nanoparticles, nanotubes, nanowires, nanorods, nanosheets, nanofibers, etc.). Over the course of the many decades that have passed, there has been a steadily expanding interest in nanofibers derived from all of these nanostructure systems. The sensitivity of the electrode that is used in the design of the sensor is an important factor that plays a role in how well biosensors fulfill their functions. In recent years, electrodes made of carbon-based nanomaterials like carbon nanotubes, graphene, and carbon nanofibers (CNFs) have been employed as substrates and protectors to increase the electrical conductivity of electrodes [65,66]. Examples of these nanomaterials include carbon nanotubes and graphene. Because of the presence of conductive carbon nanomaterials and a very high specific surface area, the biosensor is able to enhance charge transfer and quick electron transfer, which in turn boosts its sensitivity [67]. Conducting polymers, particularly polyaniline, have proven to be useful in the field of sensors due to the ease with which they can be synthesized, the doping and de-doping behavior they exhibit, the ease with which they can be deposited on substrates, and their reactivity towards gases. Doping will raise the conductivity range while maintaining a high degree of reversibility. Because of this property, doping is commonly utilized in the detection of harmful gases like CO, NO_2_, and others [68]. In this review, we discussed the recent development Electrospun nanofibers for sensors and biosensors applications.

### 4.1. Glucose Sensor

**Figure 15:**
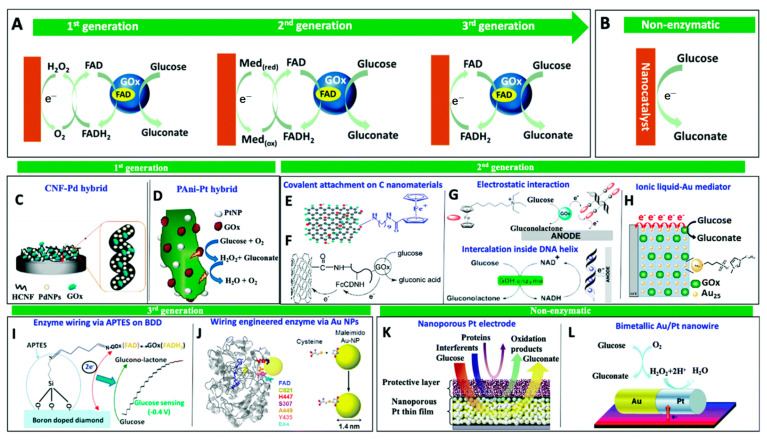
The evaluation of glucose sensor materials by various types of materials (**A**–**L**) [10].

Glucose is most importantly diagnosed in the human body for diabetes management, bioprocess monitoring, food industries, and environmental monitoring, so it is necessary to scale or presence of glucose sensor. Three generations of enzymatic glucose biosensors have been developed (Figure 15). The first generation used an oxidation reaction involving glucose, reaction mediators, and enzymes to produce gluconic acid and H_2_O_2_. The second generation used nanomaterials to slow down the rate at which enzymes leach out of a system and ensure that sensors can be reproduced accurately. Nanostructures play an important role in the third-generation biosensor electrodes, and the high surface area of nanofibers enables better action between the electrode and the enzyme. However, these biosensors are not stable over the long term, and changes in pH, temperature, and time have a significant impact on their accuracy. To address the limitations of enzyme biosensors, more focus has been placed on the research and development of enzyme-independent glucose sensors, also known as non-enzymatic glucose sensors. Substances that may electrochemically react at the sensor’s working electrodes can interfere with glucose sensors, and the results of the reaction’s generator can serve as interference detection indicators. The sensor itself can also identify interference.

**Figure 16:**
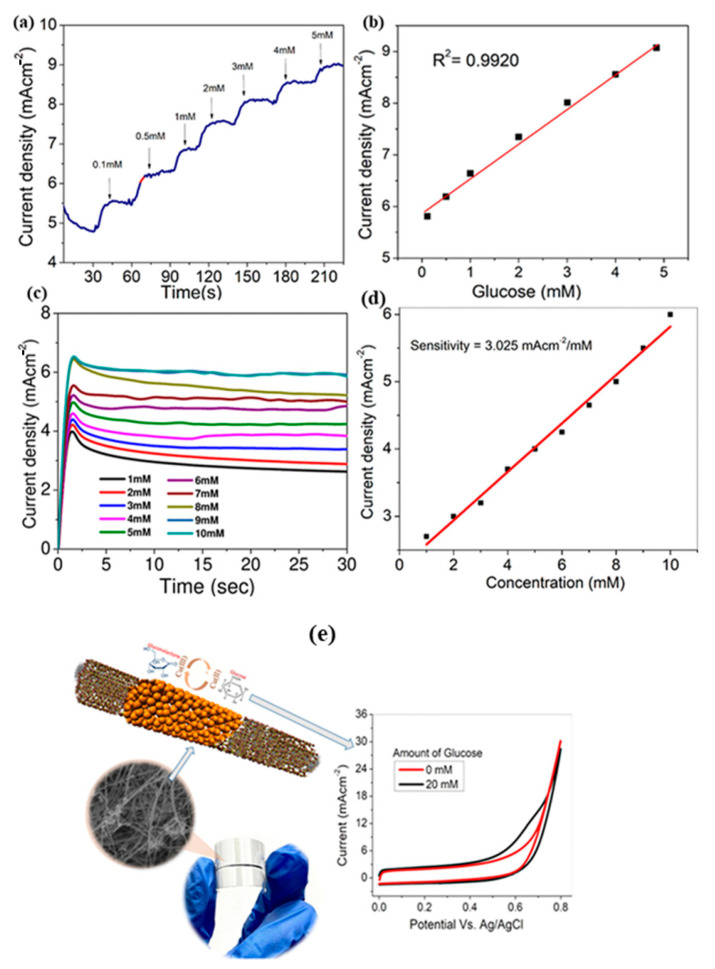
The stair behavior of the amperometry curve of the electrode material (**a**); calibration curve (**b**); (**c**) straight amperometry property on different concentrations of glucose and (**d**) its calibration curve; the schematic representation on glucose sensor by CNT–CuO nanofiber (**e**).

The fiber-like electrode materials prepared a glucose sensor by using the electrochemical method. The fiber structured composite materials have been prepared by the doping of CuO on the surface of the CNTs (Figure 16). The CuO substance has been coated on the surface of the CNTs by environmentally friendly electrodeposition method. Also, the advantage of the electro deposition involves the uniform deposition of the CuO on the surface of the CNTs. The amperometry and voltammetry analysis implies the detection range of glucose with sensitivity of ~3000 μA/mM cm^−2^, with the lower detection levels of 1.4 μM and the wide linear range of up to 13 mM. The sensing of the glucose mechanism is due to the synergetic effect between the CuO and CNTs. The resistance of the material is much lower when compared to the starting materials, which enhances the total conductivity of the material. The obtained material possesses high mechanical strength and good conductivity in nature. The advantages of these CuO–CNTs include as highly flexible and much comfortable for the wearable sensor towards the glucose [70].

### 4.2. Uric Acid Sensor

**Figure 17:**
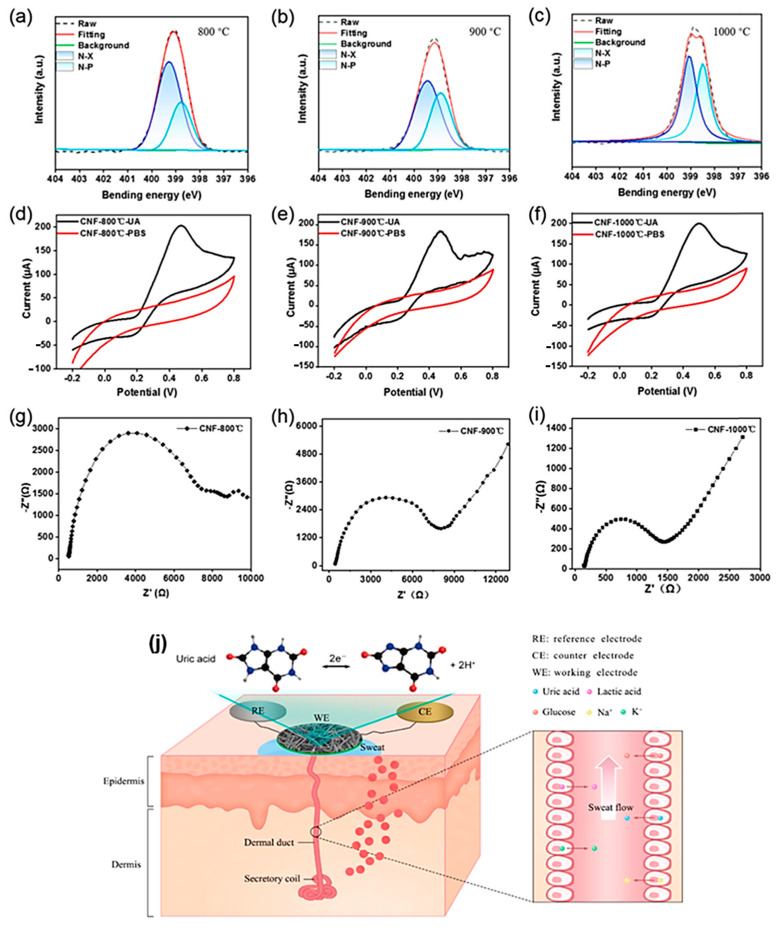
The XPS N1s spectra (**a**–**c**) of CNF on different carbonization temperature; the electro-chemical results on the uric acid sensor by Electrospun nanocomposite (**d**–**i**); the schematic working mechanism (**j**) of the wearable device.

The polyacrylonitrile (PAN) was used to detect the detection of uric acid as follows: the PAN was first made as fiber type materials by using the electrospinning method (Figure 17). The material next stabilized by different temperatures of 200, 220, 250, and 280 °C for two hours. Finally, the carbon nanofiber structured was obtained by the carbonization of those materials with different temperature of 800, 900, and 1000 °C in N_2_ atmosphere. The prepared CNF materials were tested against the uric acid detection which is present in artificial sweets. Among these materials, the PAN-1000 shows the better efficiency in terms of better conductivity and graphitized carbon structure [71].

### 4.3. Cholesterol Sensor

**Figure 18:**
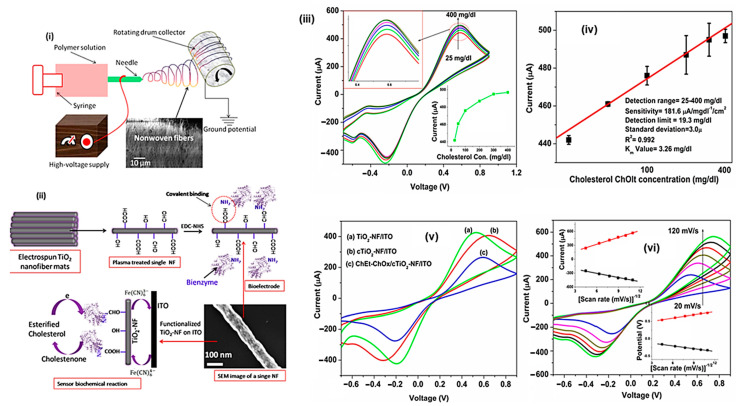
(**i**) Schematic representation of electrospinning preparation (**i**) and cholesterol sensing mechanism (**ii**) of the TiO_2_-NF; (**iii**) CV result and (**iv**) electrochemical current vs. logarithm of cholesterol ChOlt concentration plot of the nanofiber material; CV analysis (**v**) on various nanofiber materials and various scan rate (**vi**) results of the prepared nanofiber materials.

The titania-based nanofiber (TiO_2_-NF) was used for the detection of esterified cholesterol molecules by the electrochemical sensor method. The TiO_2_-NF was prepared by the electro spinning technique and further calcinated at 470 °C to obtain the nanofiber structure material. The surface of the carbonized fiber material was further functioned by using plasma treatment method. The surface functionalized materials contain -COOH, -CHO on the surface. The high mesoporous material (61%) allows the enzymatic molecules reaction on the surfaces. The result suggests that the materials show sensitive and fast detection of the esterified cholesterol molecules (Figure 18). The prepared materials could be the potential candidate for the biosensor application [72].

The information in Table 1 represents the recent trends of various materials towards the sensing of hazardous materials by electrochemical method [73,74,75,76,77,78,79,80,81,82,83,84,85,86,87,88,89,90,91,92].

**Figure 19:**
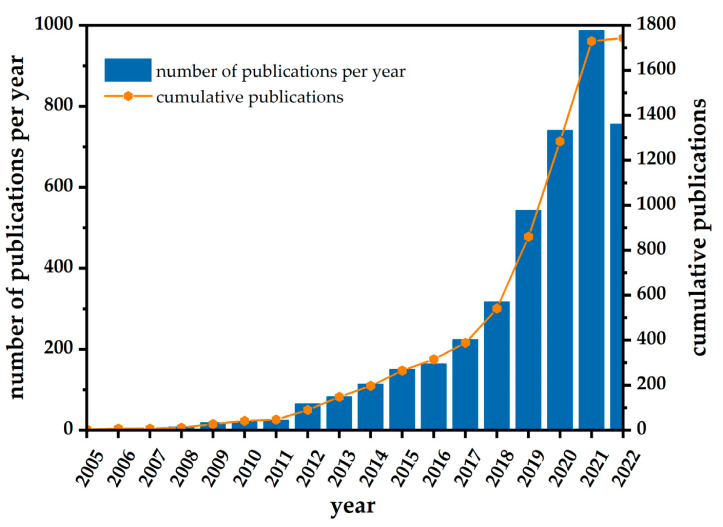
Number of publications per year on nano-silver based electrochemical sensor for environmental analysis.

Figure 19 replicates the recent research and development on the silver nanoparticles towards the electrochemical sensor method. The major challenges of the electrochemical sensor include the materials’ stability and recyclability upon the real sample analysis. To overcome these issues of the materials’ compositions, experimental and electrospinning conditions are optimized several times to obtain the targeted material.

## 5. Conclusions

In conclusion, the proposed review article discussed the details of Electrospun methods towards the sensor application. The efficient electrochemical method used to detect the different types of analytes reported in recent research was discussed in lucid manner. The chemical and biosensors have become an essential tool in various applications; the present review article emphasized the methods and importance of electrochemical sensors using Electrospun nanofiber materials. The ability to accurately detect and quantify a wide range of analytes has revolutionized diagnostics, and biosensors have shown potential in personalized medicine and remote healthcare applications. With the integration of electrochemical sensors with mobile devices and wireless communication technologies, real-time monitoring has become possible, leading to better patient outcomes. (future focuses should be on): The counterargument could be that while electrochemical sensors have shown potential in various fields, there are still limitations to their accuracy and sensitivity, especially when it comes to detecting low concentrations of analytes or dealing with complex matrices such as blood or urine. Additionally, the cost of developing and manufacturing electrochemical sensors can be high, making them less accessible for certain applications and regions. However, despite their potential, electrochemical sensors can provide significant challenges in terms of stability, selectivity, and sensitivity.

## Figures and Tables

**Table 1:** Electrospun fabricated materials for various types of sensor applications.

S. No	Material	Sensor	Method	Detection Limit	Ref.
1	WS_2_/WO_3_ heterojunctions	Acetone	In situ oxidation technique	20 ppm	[73]
2	A/γ-Fe_2_O_3_ is coupled with MXene (Ti_3_C_2_Tx)	Acetone	Sol-gel method	0.5 ppm	[74]
3	Heterojunctions of Ta_2_O_5_ and multiwalled carbon nanotubes (MWCNTS)	Ethanol	Hydrothermal method	0.173 ppm	[75]
4	Vanadium oxide nanobelts	Ethanol	Hydrothermal	5 ppm	[76]
5	Urea-modified zinc oxide thin films	Ammonia	Spray pyrolysis	25 ppm	[77]
6	Polypyrrole/functionalized MWCNT	Ammonia	Chemical oxidative polymerization method	5 ppm	[78]
7	Phosphorus-doped graphene	Ammonia	Chemical vapor deposition (CVD)	0.068 ppm	[79]
8	Hydrogel (Dimethylaminopropyl methacrylamide, methyl methacrylate, and 2-hydroxyethyl methacrylate)	CO_2_	Soft-lithographic duplication of photoinduced surface relief-gratings	2 ppm	[80]
9	Polyethylenimine (PEI) layer	CO_2_	Colloidal etching lithography/ spin coating	1 ppm	[81]
10	Ag nanoparticles/ RGO modified ITO	H_2_O_2_	Laser-scribing method	111.18 ppm	[82]
11	Ti_3_C_2_tx/pt-pd	H_2_O_2_	Microfluidic approach	10.20 ppm	[83]
12	PEDOT:PSS	H_2_O_2_	In situ electrochemical polymerization	1.0 ppm	[84]
13	Ti_3_C_2_T_x_/WS_2_	NO_2_	Sonication method	0.011 ppm	[85]
14	Two-dimensional WSe_2_	NO_2_	Selenization of tungsten trioxide	<0.1 ppm	[86]
15	A Cu complex of porous organic polymer based on porphyrin (Cu-TEG-POR)	Glucose	Polymer synthesis/complexation	162.14 ppm	[87]
16	N-Doped Co_3_O_4_ Nanoparticles/graphene	Glucose	Laser-scribing technique	86.47 ppm	[88]
17	N-doped reduced graphene oxide/au dual aerogels	Uric Acid	Hydro- and Aerogel Synthesis	622.01 ppm	[89]
18	B-doped graphene quantum dots anchored to carbon nanotubes	Uric acid	Hydrothermal/ synthesis	166.43 ppm	[90]
19	Tio_2_ nanowire bridged 3D graphene Nano stacks	Cholesterol	Electrospinning/sonication	2319 ppm	[91]
20	Oxidized Zn–In nanostructures	Cholesterol	Co-electrodeposition /hydrothermal oxidation	38.66 ppm	[92]

## Data Availability

The data pertaining to this report can be provided on reasonable request.
